# Catastrophic expenditure and impoverishment after caesarean section in Sierra Leone: An evaluation of the free health care initiative

**DOI:** 10.1371/journal.pone.0258532

**Published:** 2021-10-15

**Authors:** Alex J. van Duinen, Josien Westendorp, Thomas Ashley, Lars Hagander, Hampus Holmer, Alimamy P. Koroma, Andrew J. M. Leather, Mark G. Shrime, Arne Wibe, Håkon A. Bolkan

**Affiliations:** 1 Institute of Clinical and Molecular Medicine, Norwegian University of Science and Technology (NTNU), Trondheim, Norway; 2 Department of Surgery, St Olav’s Hospital, Trondheim University Hospital, Trondheim, Norway; 3 Kamakwie Wesleyan Hospital, Kamakwie, Sierra Leone; 4 Department of Surgery, Connaught Hospital, Freetown, Sierra Leone; 5 Centre for Surgery and Public Health, Clinical Sciences Lund, Skåne University Hospital, Lund University, Lund, Sweden; 6 Department of Global Public Health, Karolinska Institutet, Stockholm, Sweden; 7 Ministry of Health and Sanitation, Freetown, Sierra Leone; 8 Department of Obstetrics and Gynaecology, Princess Christian Maternity Hospital (PCMH), University Teaching Hospitals Complex, University of Sierra Leone, Freetown, Sierra Leone; 9 King’s Centre for Global Health & Health Partnerships, King’s College London, London, United Kingdom; 10 Department of Global Health and Population, Harvard School of Public Health, Boston, MA, United States of America; University of Waterloo, CANADA

## Abstract

**Background:**

Utilizing surgical services, including caesarean sections, can result in catastrophic expenditure and impoverishment. In 2010, Sierra Leone introduced the Free Health Care Initiative (FHCI), a national financial risk protection program for the most vulnerable groups. Aim of this study was to investigate catastrophic expenditure and impoverishment related to caesarean section in Sierra Leone and evaluate the impact of the FHCI.

**Methods:**

Women who delivered by caesarean section in nine hospitals were followed up with home visits one month after surgery, and data on medical and non-medical expenditures were collected. Individual income was estimated based on household characteristics and used to determine catastrophic expenditure and impoverishment for each patient. The impact of the FHCI was assessed by comparing actual expenditure with counterfactual expenditures had the initiative not existed.

**Results:**

For the 1146 patients in the study, the median expenditure was 23 (IQR 4; 56) international dollars (Int$). Patients in the poorest quintile spent a median Int$ 59 (IQR 28; 76), which was significantly more than patients in the richest quintile, who spent a median Int$ 17 (IQR 2; 38, p<0.001). Travel (32.9%) and food (28.7%) were the two largest expenses. Catastrophic expenditure was encountered by 12.0% and 4.0% (10% and 25% threshold, respectively) of the women. Without the FHCI, 66.1% and 28.8% of the women would have encountered catastrophic expenditure.

**Conclusion:**

Many women in Sierra Leone face catastrophic expenditure related to caesarean section, mainly through food and travel expenses, and the poor are disproportionally affected. The FHCI is effective in reducing the risk of catastrophic expenditure related to caesarean section, but many patients are still exposed to financial hardship, suggesting that additional support is needed for Sierra Leone’s poorest patients.

## Introduction

### Catastrophic expenditure and impoverishment

Surgery has been acknowledged as an integral component of universal health coverage [1]. Scaling up surgical services is necessary to provide the additional 143 million procedures needed each year to save lives and prevent disability [2]. Surgery is considered highly cost-effective [3], nevertheless, utilizing surgical services can have negative financial consequences for individuals owing to high out-of-pocket expenditure [4]. Reflecting the importance of financial risk protection for surgical care, two of the six indicators from the Lancet Commission on Global Surgery to measure surgical care at the global level are dedicated to financial consequences: catastrophic expenditure and impoverishment [5].

Catastrophic expenditure occurs when expenditure related to treatment surpasses 10% or 25% of annual income, as defined by the Sustainable Development Goals-monitoring framework and adopted by the World Bank and World Health Organization [6]. An estimated 81 million people face catastrophic expenditure every year because of expenses related to surgical care [4]. Impoverishment takes place when treatment-related spending pushes individuals below the poverty line of 1.90 international dollars (Int$) per day [6, 7]. It is estimated that between 31% and 57% of the world’s population is at risk of impoverishing expenditure [8].

The risk of adverse economic outcomes because of surgery is the highest in sub-Saharan Africa [8], where a large proportion of healthcare is financed out-of-pocket [9]. The World Bank has adopted the risk of catastrophic expenditure and impoverishment as one of its World Development Indicators [7], but few countries have been able to supply updated and detailed data [10].

### Maternal health and caesarean section

Caesarean section is one of the most commonly performed surgical procedures worldwide and national caesarean section rates vary between 0.6% in South Sudan and 58.1% in the Dominican Republic [11]. A caesarean section rate under 9–19% has been associated with poor maternal and neonatal outcomes [12, 13]. Approximately two-thirds of the maternal deaths in low-income countries can be avoided if caesarean section rates rise to 10–15%, as recommended by the WHO [14]. In these settings, financial constraints are a major barrier for patients seeking and accessing emergency obstetric care [15]. In addition, the risk for catastrophic expenditure is 2–7 times higher after caesarean section versus vaginal delivery [16, 17].

### Sierra Leone

Sierra Leone has one of the world’s highest maternal mortality ratios of 1165 deaths per 100,000 live births [18], and a caesarean section rate of 2.9% [19]. The country is still recovering from civil war (1991–2002) and the Ebola epidemic (2014–2016) [20], and has a fragile health system in which the majority (62%) of health expenditure is paid for out-of-pocket [21]. The gross domestic product (GDP) per capita is roughly Int$ 1600 and more than 60% of the population lives under the poverty line of Int$ 1.90 a day [7].

In 2010, the government of Sierra Leone introduced the Free Health Care Initiative (FHCI), which made health services free for all pregnant and lactating women as well as children under five [22]. All government-operated healthcare facilities take part in the FHCI, while private non-profit healthcare facilities are encouraged to participate as well to supplement the services provided by the public sector. The FHCI abolished user fees with the intention of offering protection against catastrophic expenditure and impoverishment. Although the positive effects of user fee exceptions have been widely debated, the FHCI in Sierra Leone, has been successful in increasing the number of institutional deliveries and antenatal care visits, thereby promoting equity [23–25]. However, little is known about the initiative’s impact on catastrophic expenditure and impoverishment related to caesarean section.

This study aims to estimate the proportion of women who face catastrophic expenditure and impoverishment related to caesarean section and evaluate the impact of the FHCI on rates of financial hardship.

## Methods

### Data

This study was part of a prospective observational multicentre study of women undergoing caesarean section in Sierra Leone [26]. A total of nine hospitals were included in this study, four district hospitals, one regional hospital, the national maternity referral hospital, and three private non-profit hospitals. At each hospital, anaesthesia team members were trained to enrol patients in the study and collect patient data after written informed consent was obtained by signature or thumbprint, either before or as soon as possible after surgery. The primary investigator compiled and reviewed the data during regular hospital visits at one- to three-week intervals. For each caesarean section, data were entered in an Excel database (Microsoft Corp., Redmond, USA). Missing or inconsistent data were supplemented from operation logbooks or patient files. Four trained research nurses made follow-up home visits one month after caesarean section, during which information on the educational level and marital status of the mother, and medical (admission, consultation and medication) and non-medical (travel, lodging and food) expenses were collected. In addition, information on household characteristics were captured through questions and observations based on the 2013 Sierra Leone Demographic and Health Survey (DHS-SL13) methodology [18].

**Fig 1:**
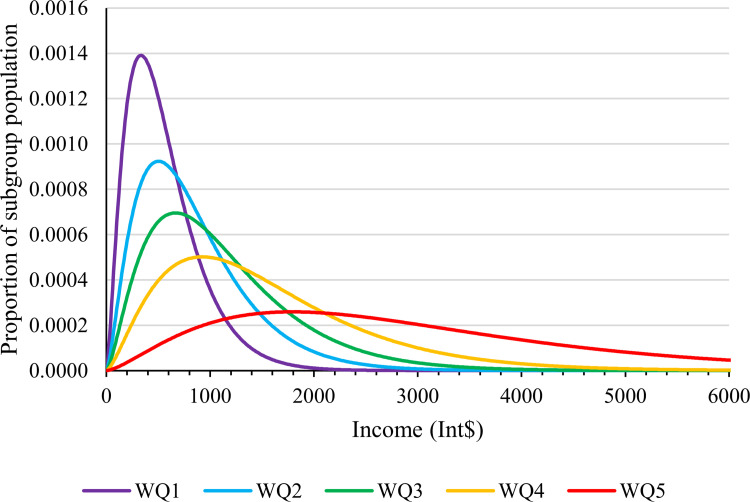
Income distribution by wealth quintile. Graphical representation of estimated household income with each curve representing a different wealth quintile (WQ). These gamma distributions are based on the Sierra Leonean Gross Domestic Product (GDP) per capita (purchasing power parity, constant 2011 international dollars) from 2017, income share per wealth quintile, and the GINI coefficient (data.worldbank.org).

Households were assigned to wealth quintiles based on the household characteristics and scores established by the principal component analysis of the DHS-SL13 [18]. Mean annual income was estimated for each wealth quintile based on the 2017 GDP per capita in 2011 constant Int$ and income share per wealth quintile [27]. A previously described method of gamma distribution based on the national GINI coefficient was employed to represent the distribution of individual incomes for each wealth quintile (Fig 1) [8, 28], and a random selection step was utilised to assign individual annual household incomes to each patient [29].

All medical and non-medical expenses were recorded in the local currency, Sierra Leone Leones (SLL), and adjusted for time by dividing by the 2011–2017 SLL deflation correction of 1.69 [27, 30]. Subsequently, tradable expenses (medication and food) were converted to Int$ by applying the 2011 market exchange rate of 4349 and non-tradable expenses (admission, consultation, travel, and lodging) were converted to Int$ using the 2011 purchasing power parity (PPP) conversion rate of 1553 [27, 30].

Catastrophic expenditure was defined as total expenses exceeding a set proportion of annual household income using the internationally established thresholds of 10% and 25% [31]. Impoverishing expenditure was defined as expenditure that pushed individuals under the poverty line of Int$ 1.90 a day (or Int$ 694 per year). Finally, the catastrophic expenditure rate was adjusted for the population by using the wealth quintile-specific caesarean section rates from the DHS-SL13 [18].

To study the impact of the FHCI, a counterfactual scenario was generated, in which patients would have to pay for their caesarean section. As patient fees for caesarean sections have been abolished since 2010, the patient fee for a laparotomy (ranging from Int$ 190 and Int$ 571, dependent on the hospital) was used as a proxy for the cost of a caesarean section and added to total expenses to simulate a situation without the FHCI. The price of a laparotomy was selected as a proxy for the price of a caesarean section as the fees for these two procedures were the same (SLL 200,000 / Int$ 129) before the implementation of the FHCI and are still comparable in the private for-profit health sector in Sierra Leone, which does not participate in the FHCI [32]. The impact of the FHCI in terms of protection against catastrophic expenditure is defined as the number of patients who would have faced catastrophic expenditure without the FHCI minus the number who actually faced catastrophic expenditure divided by the number who would have faced catastrophic expenditure without the FHCI. The same calculations were performed for impoverishment. National estimates were obtained by applying weighting factors from the distribution of caesarean sections over hospital categories [32]. The methodology has been described in more detail in the supporting information (S1 File).

### Statistical analysis

Descriptive statistics provide an overview of the study population and indicate the proportion of patients facing catastrophic and impoverishing expenditure. Normally distributed data are presented with means and a 95% confidence interval (95% CI), and non-normally distributed data are presented with the median and interquartile range. Missing data are presented in tables. Statistical analyses were carried out with STATA 16.0 (StataCorp LLC, College Station, USA).

### Ethics approval

The study was approved by the Sierra Leone Ethics and Scientific Review Committee (19 May 2016) and the Regional Committees for Medical and Health Research Ethics in Central Norway (Ethical Clearance No. 2016/1163), and is registered in the International Clinical Trials Registry (ISRCTN: 16157971).

## Results

**Fig 2:**
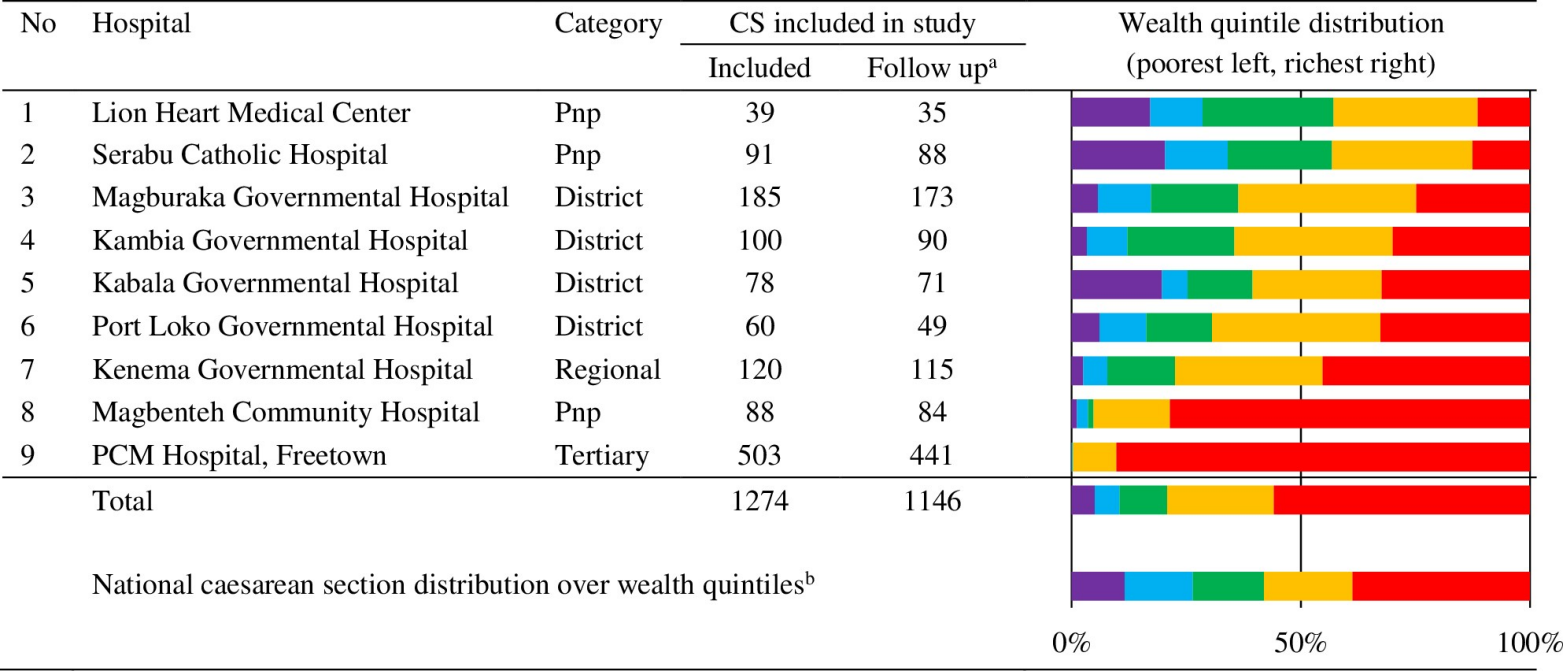
Study hospitals by category, patient inclusion and wealth quintile distribution. ^a^Follow-up with complete data and included in the analysis. ^b^2013 Sierra Leone Demographic and Health Survey. Pnp = private non-profit.

Between 1 October 2016 and 5 May 2017, 1728 caesarean sections were performed in the study hospitals, of which 73.7% (n = 1274) were included in the study [26]. Of the included patients, 91.1% (n = 1161) were followed up with home visits after one month. For 90.0% (n = 1146) of the patients, housing characteristics and expenditures data were collected included in the analysis (Fig 2).

Based on the household characteristics, the women were classified into wealth quintiles (Fig 2). Most women (n = 640; 55.8%) were placed in the richest quintile while only few (58; 5.1%) were placed in the poorest quintile. The median expenses related to caesarean section were Int$ 23 (IQR 4; 56) (Table 1). Patients in the poorest quintile spent a median Int$ 59 (IQR 28; 76), which is significantly more than patients in the richest quintile who spent a median Int$ 17 (IQR 2; 38, p<0.001). Travel (32.9%) and food (28.7%) accounted for over 60% of the total expenditure (S1 Table).

**Table 1:** Income and medical and non-medical expenses by wealth quintile.

Wealth Quintile	n	Assigned income^a^	Medical expenses^a^	Non-medical expenses^a^	Total expenses^a^
		Median (IQR)	Median (IQR)	Median (IQR)	Median (IQR)
1	58	507 (306; 826)	0 (0; 2)	45 (23; 65)	59 (28; 76)
2	62	590 (373; 929)	0 (0; 27)	26 (11; 45)	35 (16; 79)
3	119	916 (601; 1342)	0 (0; 27)	50 (2; 105)	36 (13; 84)
4	267	1311 (856; 2002)	0 (0; 13)	23 (2; 61)	28 (11; 62)
5	640	2528 (1605; 3786)	0 (0; 0)	20 (2; 44)	17 (2; 38)
Total	1146	1666 (915; 2939)	0 (0; 0)	14 (1; 29)	23 (4; 56)

^a^in international dollars.

n = number.

IQR = interquartile range.

Of all 1146 patients, 12.0% (n = 138) spent more than 10% of their annual household income, and 4.0% (n = 46) spent over 25% (Table 2). Such catastrophic health expenditure was 15.0 (95% CI 9.3–24.3) and 143.4 (95% CI 19.1–1077.2) times more common in the poorest quintile than in the richest quintile for the 10% and 25% thresholds, respectively. Applying the national distribution of caesarean sections over hospital categories [32], reveals a national rate of catastrophic expenditure for women undergone caesarean sections of 12.9% and 4.2% for the 10% and 25% thresholds, respectively. It was estimated that 17.5% (n = 200) of participating women were living below the poverty line before their caesarean section and an additional 1.1% (n = 13) became impoverished as a result of expenditure related to the procedure (Table 3).

**Table 2:** Catastrophic expenditure after caesarean section and impact of the free health care initiative.

Wealth quintile	n	Catastrophic expenditure (10% threshold)			Catastrophic expenditure (25% threshold)		
		With FHCI	Protected by FHCI^a^, n (%)	Without FHCI, n (%)	With FHCI	Protected by FHCI^a^, n (%)	Without FHCI, n (%)
		n (%)			n (%)		
1	58	30 (51.7%)	28 (48.3%)	58 (100.0%)	13 (22.4%)	38 (74.5%)	51 (87.9%)
2	62	21 (33.9%)	40 (65.6%)	61 (98.4%)	7 (11.3%)	40 (85.1%)	47 75.8%)
3	119	30 (25.2%)	85 (73.9%)	115 (96.6%)	15 (12.6%)	48 (76.2%)	63 52.9%)
4	267	35 (13.1%)	190 (84.4%)	225 (84.3%)	10 (3.7%)	78 (88.6%)	88 (33.0%)
5	640	22 (3.4%)	276 (92.6%)	298 (46.6%)	1 (0.2%)	80 (98.8%)	81 (12.7%)
Total	1146	138 (12.0%)	619 (81.8%)	757 (66.1%)	46 (4.0%)	284 (86.1%)	330 (28.8%)
National estimates^b^		12.9%	81.1%	68.1%	4.2%	86.2%	30.5%

Catastrophic expenditure after caesarean section in Sierra Leone with and without the Free Health Care Initiative (FHCI). The current situation with the FHCI and the counterfactual situation without the FHCI were simulated by adding the price of a laparotomy to the total expenditure. Catastrophic expenditure is determined by exceeding the 10% and 25% threshold of total income.

^a^percentage protected by the FHCI is based on the number of women protected against catastrophic expenditure by the FHCI divided by those that would experience catastrophic expenditure without the FHCI.

^b^weighting factors from the distribution of caesarean sections over hospital categories (Lindheim-Minde *et al*^32^).

**Table 3:** Impoverishment after caesarean section and the impact of the free health care initiative.

Wealth quintile			n	Impoverished before exp		Impoverished after exp with FHCI		Protected by FHCI^a^		Impoverished without FHCI	
				n	%	n	%	n	%	n	%
1			58	40	69.0%	41	70.7%	8	16.3%	49	84.5%
2			62	37	59.7%	38	61.3%	9	19.1%	47	75.8%
3			119	40	33.6%	46	38.7%	17	27.0%	63	52.9%
4			267	46	17.2%	49	18.4%	31	38.8%	80	30.0%
5			640	37	5.8%	39	6.1%	27	40.9%	66	10.3%
Total			1146	200	17.5%	213	18.6%	92	30.2%	305	26.6%
National estimates^b^					18.6%		19.8%		29.8%		28.2%

Impoverishment after caesarean section in Sierra Leone with and without the Free Health Care Initiative (FHCI). The current situation with the FHCI, and the situation without the FHCI were simulated by adding the price of a laparotomy to total expenditure. Impoverishing expenditure was determined by crossing below the poverty line of 1.90 international dollars a day.

^a^percentage protected by the FHCI is based on the number of women experiencing impoverishing expenditure with the FHCI divided by those that would experience impoverishing expenditure without the FHCI.

^b^weighting factors from the distribution of caesarean sections over hospital categories (Lindheim-Minde *et al*^32^).

**Fig 3:**
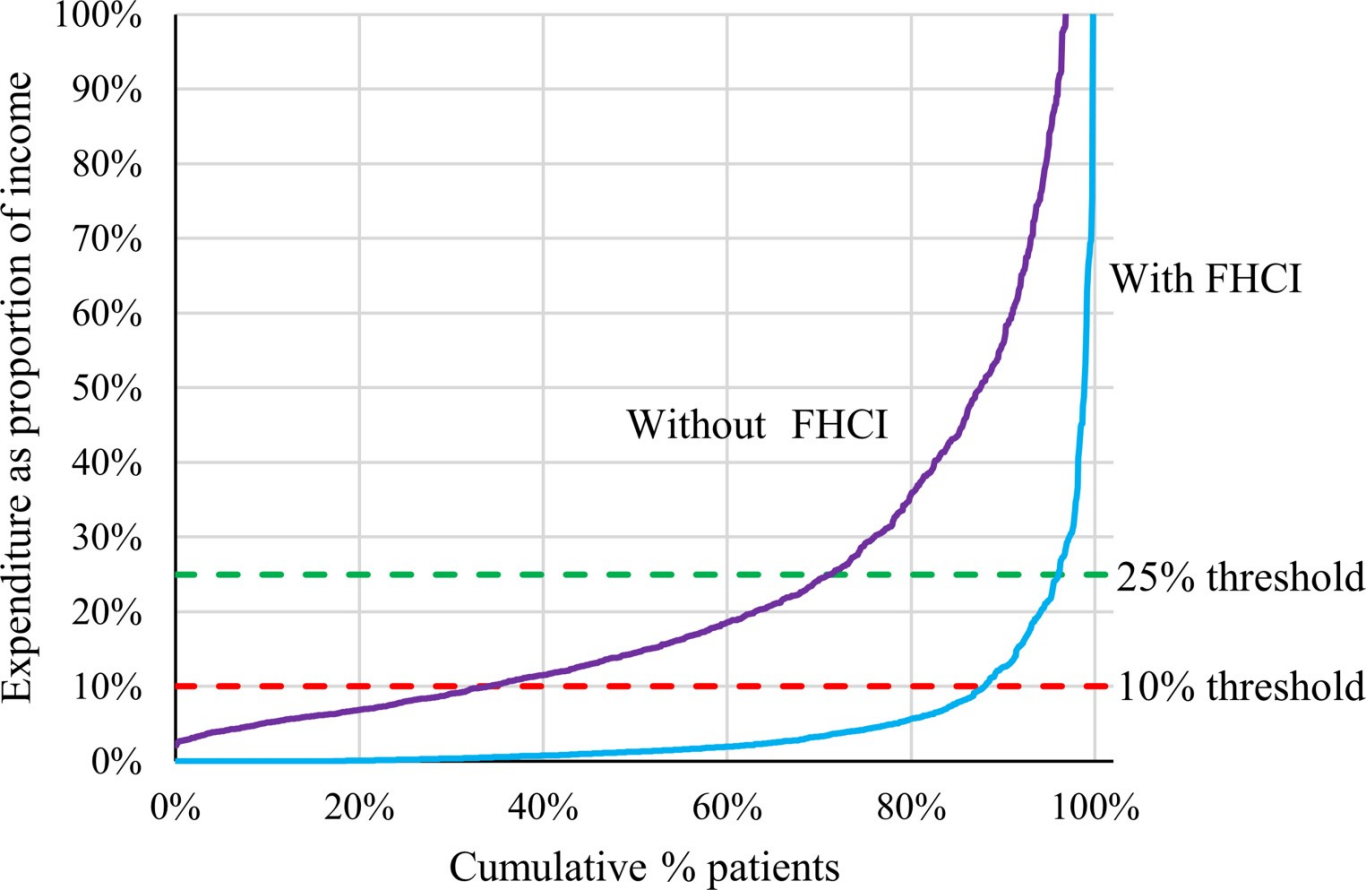
Expenditure in relation to the 10 and 25% thresholds for catastrophic expenditure. Graphical representation of the cumulative percentage of patients. The red dotted line represents the 10% threshold, and the green dotted line the 25% threshold of catastrophic expenditure. The blue line represents the current situation with the Free Health Care Initiative (FHCI) and the purple line represents the scenario without the FHCI.

In a scenario without the FHCI, with the cost of the procedure added to patients’ total expenditure, 66.1% (n = 757) and 28.8% (n = 330) of the women would have experienced catastrophic expenditure at the 10% and 25% thresholds, respectively (Fig 3). Depending on the threshold, the FHCI protected 81.8–86.1% of patients against catastrophic expenditure. National estimates for protection against catastrophic expenditure for women undergone caesarean sections were 81.1% and 86.2% for the 10% and 25% thresholds, respectively. As a consequence of the FHCI, a greater proportion of women in the richest quintile (92.6% and 98.8%) were able to avoid catastrophic expenditure compared to the poorest quintile (48.3% and 74.5%).

**Fig 4:**
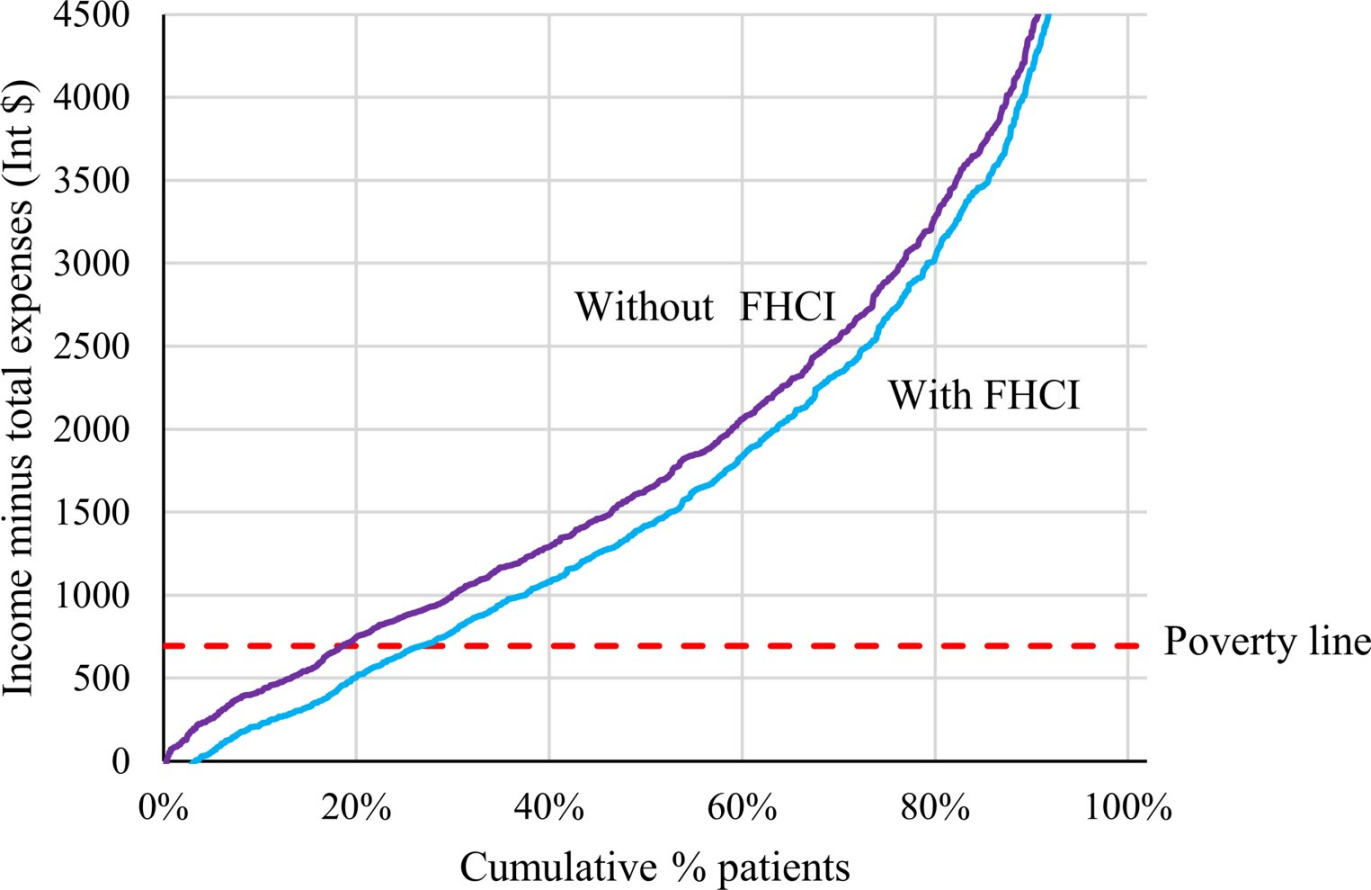
Income minus total medical and non-medical expenses in relation to the poverty line. Graphical representation of annual income minus the total of medical and non-medical expenses in international dollars (Int$). The red dotted line represents the poverty line of Int$ 1.90 per day (or Int$ 694 international dollars annually). The blue line represents the current situation with the Free Health Care Initiative (FHCI) and the purple line represents the scenario without the FHCI.

Without the existence of the FHCI, an additional 92 women would have been pushed below the poverty line (Fig 4). Therefore, the FHCI protected 30.2% (92 of 305) of women against impoverishment in this study. The national estimate for protection against impoverishing expenditure was 29.8%.

Patients with higher education spent about half the amount (Int$ 15, IQR 2; 35) than those with no education (Int$ 31, IQR 11; 68) (Table 4). Patients paid the most in district hospitals (Int$ 45, IQR 20; 79) followed by private non-profit facilities (Int$ 26, IQR 11; 54) and national referral hospitals (11, IQR 0; 30). Patients who required a blood transfusion or encountered maternal or perinatal death had higher expenses and were more likely to experience catastrophic expenditure and impoverishment.

**Table 4:** Total expenditure, catastrophic expenditure, and impoverishment by group.

	Patients	Expenditure	Catastrophic expenditure 10% threshold n (%)	Catastrophic expenditure 25% threshold n (%)	Impoverishment
	n	median (IQR)^a^			n (%)
**Social factors**					
Women’s education mother					
None	449	31 (11; 68)	89 (19.8%)	29 (6.5%)	125 (27.8%)
Primary	148	24 (5; 57)	17 (11.5%)	8 (5.4%)	30 (20.3%)
Secondary	428	18 (2; 40)	30 (7.0%)	9 (2.1%)	55 (12.9%)
Higher education	121	15 (2; 35)	2 (1.7%)	0 (0.0%)	3 (2.5%)
Marital status					
Never married	240	20 (2; 42)	19 (7.9%)	3 (1.3%)	38 (15.8%)
Married	904	23 (5; 57)	119 (13.2%)	43 (4.8%)	174 (19.3%)
Widowed	2	39 (26; 52)	0 (0.0%)	0 (0.0%)	1 (50.0%)
Age group					
< 15 years	9	11 (4;66)	1 (11.1%)	0 (0.0%)	2 (22.2%)
15–19 years	208	24 (5; 60)	35 (16.8%)	16 (7.7%)	56 (26.9%)
20–24 years	271	20 (4; 47)	22 (8.1%)	9 (3.3%)	34 (12.3%)
25–29 years	315	22 (3; 55)	34 (10.8%)	9 (2.9%)	55 (17.5%)
30–34 years	195	23 (7; 54)	22 (11.3%)	7 (3.6%)	32 (16.4%)
35–39 years	126	27 (8; 59)	23 (18.3%)	4 (3.2%)	31 (24.6%)
≥ 40 years	22	10 (24; 46)	1 (4.6%)	1 (4.6%)	3 (13.66%)
**Travel & hospital factors**					
Referred from another facility					
Referred	514	19 (4; 45)	58 (9.2%)	15 (2.4%)	97 (15.4%)
Not referred	532	28 (4; 63)	80 (15.6%)	31 (6.0%)	116 (22.6%)
Mode of transport					
Boat	5	61 (29; 145)	2 (40%)	1 (20.0%)	3 (60.0%)
Ambulance	421	34 (13; 69)	79 (18.8%)	28 (6.7%)	107 (25.4%)
Private car	24	13 (0; 41)	0 (0.0%)	0 (0.0%)	0 (0.0%)
Taxi	271	13 (3; 34)	9 (3.3%)	1 (0.4%)	23 (8.5%)
Motorbike	264	22 (6; 45)	29 (11.0%)	8 (3.0%)	52 (19.7%)
Walking	83	11 (0; 29)	3 (3.6%)	0 (0.0%)	8 (9.6%)
Other	3	3 (0; 4)	0 (0.0%)	0 (0.0%)	1 (33.3%)
Missing	75	27 (13; 71)	16 (21.3%)	8 (10.7%)	19 (25.3%)
Estimated travel time					
≤ 2 hours	920	20 (4; 48)	93 (10.1%)	25 (2.7%)	145 (15.8%)
> 2 hours	216	38 (8; 73)	44 (20.1%)	21 (9.6%)	67 (30.6%)
Missing	7	14 (0; 21)	1 (14.3%)	0 (0.0%)	1 (14.3%)
Facility category					
District hospital	383	45 (20; 79)	92 (24.0%)	36 (9.4%)	108 (28.2%)
Referral hospital	556	11 (0; 30)	18 (3.2%)	3 (0.5%)	56 (10.1%)
Private non-profit hospital	207	26 (11; 54)	28 (13.5%)	7 (3.4%)	49 (23.7%)
**Clinical factors**					
Parity					
Nullipara (para 0)	379	20 (3; 58)	38 (10.0%)	17 (4.5%)	57 (15.0%)
Multipara (para 1–4)	631	22 (5; 47)	66 (10.5%)	21 (3.3%)	109 (17.3%)
Grand multipara (para ≥ 5)	136	38 (11; 76)	34 (25.0%)	8 (5.9%)	47 (34.6%)
Urgency					
Planned	166	20 (2; 46)	20 (12.1%)	5 (3.0%)	23 (13.9%)
Emergency	980	23 (5; 57)	118 (12.0%)	41 (4.2%)	190 (19.4%)
Indication group					
Antepartum haemorrhage	130	24 (4; 46)	14 (10.8%)	7 (5.4%)	25 (19.2%)
Obstructed and prolonged labour	633	22 (4; 57)	74 (11.7%)	22 (3.5%)	116 (18.3%)
Uterine rupture	49	23 (0; 65)	11 (22.5%)	4 (8.2%)	10 (20.4%)
Foetal indication	80	20 (3; 53)	17 (21.3%)	6 (75%)	20 (25.0%)
Previous caesarean section	149	21 (2; 38)	6 (4.0%)	1 (0.7%)	19 (12.8%)
Other	105	30 (11; 69)	16 (15.2%)	6 (5.7%)	23 (21.9%)
**Outcome factors**					
Blood transfusion					
No	316	22 (5; 53)	91 (11.0%)	32 (3.9%)	150 (18.1%)
Yes	827	25 (2; 67)	47 (14.9%)	14 (4.4%)	62 (19.6%)
Missing	3	22 (16; 38)	0 (0.0%)	0 (0.0%)	1 (33.3%)
Perioperative maternal death^b^					
No	1133	22 (4; 55)	132 (11.7%)	46 (4.1%)	208 (18.4%)
Yes	13	75 (36; 91)	6 (46.2%)	0 (0.0%)	5 (38.5%)
Perinatal death					
No	214	21 (4; 52)	97 (10.4%)	33 (3.5%)	163 (17.5%)
Yes	932	29 (4; 69)	41 (19.2%)	13 (6.1%)	50 (23.4%)
**Total**	1146	23 (4: 56)	138 (12.0%)	46 (4.0%)	213 (18.6%)

^a^in international dollars.

^b^Defined as maternal death during caesarean section or within 30 days after the surgery.

n = number.

IQR = interquartile range.

## Discussion

The 1146 women in this study spent a median of Int$ 23 related to caesarean section, even though healthcare for pregnant women is free of charge in Sierra Leone. Most expenses were related to travel and food. Between 4.0% and 12.0% of the women in this study that underwent a caesarean section experienced catastrophic expenditure, at the 25% and 10% thresholds of annual household expenditure, respectively.

Women in the poorest quintile had a 15–143-fold higher risk of catastrophic expenditure compared to the richest quintile. Two other studies from sub-Saharan Africa have presented rates of catastrophic expenditure. In Mali, 33.5% (applying a 10% threshold) of women experienced catastrophic expenditure after emergency obstetric care [33], and in the Democratic Republic of Congo, 47.2% (applying a 40% capacity to pay threshold) experienced catastrophic expenditure after caesarean section [16].

Catastrophic expenditure and impoverishment can have enormous consequences for individuals and their household. It can lead to losing the ability to pay rent or school fees or even require reduced food consumption in the household [33]. In addition, financial deprivation can lead to social issues such as verbal abuse, disputes with in-laws, denial of paternity and even divorce [16].

According to our findings, the FHCI protected 81.8%–86.1% against catastrophic expenditure. Due to the FHCI, women from the richer quintiles were more effective in avoiding catastrophic expenditure compared to women form the poorest quintile. Similar observations are reported in Morocco where the Free Deliveries and Caesarean Section policy, was shown to be largely effective, although more funding was necessary to lift the financial burden carried by those in lower income groups [34]. An evaluation of the user fee exemption policies focused on caesarean sections in Benin and Mali found no substantial impact on the place of residence and socioeconomic inequalities [35]. These examples suggest that user fee exemption policies alone are not enough to reduce inequity in access to caesarean, but a more targeted approach towards financial risk protection for the poorest is essential.

Another study from Sierra Leone that evaluated the FHCI, stated that for antenatal and postnatal care, the initiative managed to reduce inequity while for institutional deliveries it was insufficient in addressing wealth-related inequity [23]. However, in the same study the authors found that for access to institutional deliveries, wealth related inequity increased after the start of the FHCI to the advantage of the rich, highly educated, and urban residents. The finding from our study that women in the highest wealth quintiles are better protected by the FHCI for catastrophic and impoverishing expenditures caesarean sections confirms the previous conclusion that the FHCI has not been able to reduce inequity for hospital-based obstetric care [23].

Transport expenses were highest in the poorest quintile. In another manuscript, based on the same database, it was found that patients in the poorest quintile had a median reported travel time that was more than double that of the richest quintile (113 versus 45 minutes) [36]. Since October 2018, a National Emergency Medical Service has been established providing free transport for obstetric patients between primary health care units (PHUs) and hospitals [37]. Emergency phone requests from PHUs are received in a centralized operation centre that coordinates the response of the total of 81 operating ambulances.

Based on the finding that the poorest have the highest travel costs, it is expected that the introduction of this free ambulance system promotes equity and is most beneficial for those that need it the most. Furthermore, providing free food for patients that are admitted might also reduce expenditures, improve accessibility, and decrease inequity.

Part of the success of the FHCI can be attributed to the parallel implementation of Human Resources for Health reforms increasing salaries and fighting absenteeism [24]. However, sustaining the momentum for reform and fulfilling increased financial commitments (increased salaries, rural allowances and performance-based financing) remains a challenge for the government of Sierra Leone [24].

Observing the positive effects of the FHCI in promoting universal health coverage for women needing caesarean section, raises the question of potential financial risk protection benefit, if the FHCI were to be expanded for patients with other conditions that require emergency surgical care. Lifting financial barriers can potentially lead to lower mortality and morbidity and promote economic growth. However, implementation of such an insurance scheme will also lead to additional expenses for the health care sector which needs to be carried by the Ministry of Health and Sanitation.

### Strengths and limitations

The main strength of this study is the prospective design with data collected from over 1100 patients from 9 different hospitals across Sierra Leone. During the home visits, one month after surgery, wealth quintiles were assigned based on standardized questionnaires and observations. Using this information to assign an annual income is likely to be more reliable than questioning participants directly about annual salary as less than 10% of the population has a fixed monthly salary [31]. However, this method, which makes use of the national GINI-coefficient, gross domestic product per capita and income share per wealth quintile, also introduces potential bias [29]. For the medical and non-medical expenses, information was purposefully recorded one month after the caesarean during home visits in a safe environment to get the best information on a potentially sensitive topic. However, this delay could have led to recall bias.

The selection of hospitals was done primarily to compare caesarean sections performed by medical doctors against clinical officers as reported elsewhere [26]. Only 1274 (73.7%) of 1728 eligible caesarean sections were included as 446 (25.8%) were not assessed for inclusion and 8 (0.5%) dropped out. This could have introduced a substantial selection bias. Reasons for patients not being assessed for inclusion included unavailable trained data collection staff or materials and clinical work being prioritized over data collection during busy periods of clinical activity.

Compared with the national distribution of wealth quintiles among those undergoing a caesarean section, there is a selection bias in our study as evident by the higher proportion of caesarean sections from the richest quintile group, (56%) compared to the DHS-SL13 (39%) [18]. This difference can be explained by the relatively large group of patients in the study that are from Western Area, which includes the capital city of Freetown. This selection bias provides a more conservative estimate for financial hardship.

This study is facility based and therefore only contains data from patients that managed to reach hospital. Women for whom the barriers were too high to seek care and reach care at the hospital level are not included in this study. Indeed, but the fact that caesarean sections are unequally distributed over the wealth quintiles suggests that this group exists. In addition, this study does not account for indirect costs or lost opportunities. Time spent in hospital can lead to loss of income and not being able to pay school fees can lead to lost educational, and in the long-run, career opportunities [16, 30].

## Conclusion

Many women are facing catastrophic expenditure related to caesarean section in Sierra Leone, mainly related to food and transport and the poorest have the highest expenses. The FHCI is effective in reducing the risk for catastrophic expenditure related to caesarean section, but many patients are still exposed to financial hardship, suggesting that additional support is needed for the poorest patients.

## Supporting information

S1 Table Distribution of medical and non-medical expenses. ^a^in international dollars. n = numbers.
(DOCX)

S1 File Methodology description. Detailed description of financial calculations.
(DOCX)
