# Supplementary material for: Catastrophic expenditure and impoverishment after caesarean section in Sierra Leone: An evaluation of the free health care initiative
Source: PLoS One. 2021 Oct 15;16(10):e0258532. doi: 10.1371/journal.pone.0258532 (PMC8519447; doi:10.1371/journal.pone.0258532)
Supplement: S1 Table — ain international dollars. n = numbers. (DOCX) [file pone.0258532.s001.docx]

**S1 Table. Distribution of medical and non-medical expenses.**

| Wealth  Quintile | n | Medical expenses^a^  Mean (%) | | | |  | Non-medical expenses^a^  Mean (%) | | | Total^a^  Mean |
| --- | --- | --- | --- | --- | --- | --- | --- | --- | --- | --- |
|  |  | Admission | Consultation | Medication | Other |  | Travel | Food | Lodging |  |
| 1 | 58 | 0 (0.0%) | 0 (0.7%) | 2 (3.9%) | 10 (16.5%) |  | 36 (58.6%) | 12 (20.2%) | 0 (0.1%) | 61 |
| 2 | 62 | 0 (0.0%) | 2 (3.7%) | 5 (10.3%) | 10 (20.5%) |  | 22 (45.3%) | 10 (20.2%) | 0 (0.0%) | 49 |
| 3 | 119 | 0 (0.0%) | 4 (6.1%) | 7 (12.2%) | 13 (20.7%) |  | 24 (40.5%) | 12 (20.3%) | 0 (0.2%) | 60 |
| 4 | 267 | 0 (0.0%) | 3 (7.4%) | 7 (14.1%) | 10 (21.6%) |  | 15 (31.5%) | 12 (25.0%) | 0 (0.4%) | 47 |
| 5 | 640 | 0 (0.9%) | 3 (8.8%) | 3 (8.9%) | 6 (19.1%) |  | 8 (25.1%) | 12 (36.4%) | 0 (0.7%) | 34 |
| Total | 1146 | 0 (0.4%) | 3 (7.1%) | 4 (10.5%) | 8 (19.9%) |  | 14 (32.9%) | 12 (28.7%) | 0 (0.4%) | 42 |

^a^in international dollars.

n = numbers.
