# Supplementary material for: Catastrophic expenditure and impoverishment after caesarean section in Sierra Leone: An evaluation of the free health care initiative
Source: PLoS One. 2021 Oct 15;16(10):e0258532. doi: 10.1371/journal.pone.0258532 (PMC8519447; doi:10.1371/journal.pone.0258532)
Supplement: S1 File — Detailed description of financial calculations. (DOCX) [file pone.0258532.s002.docx]

Catastrophic expenditure and impoverishment after caesarean section in Sierra Leone: An evaluation of the impact of the Free Health Care Initiative

*Supplementary Material - methodology description*

This document describes the methodology used to calculate catastrophic and impoverishing expenditure for women undergoing caesarean section in Sierra Leone and the impact of the Free Health Care Initiative. The process is described in 7 steps (Figure 1).

*Figure 1. Step-by-step description of financial calculations*


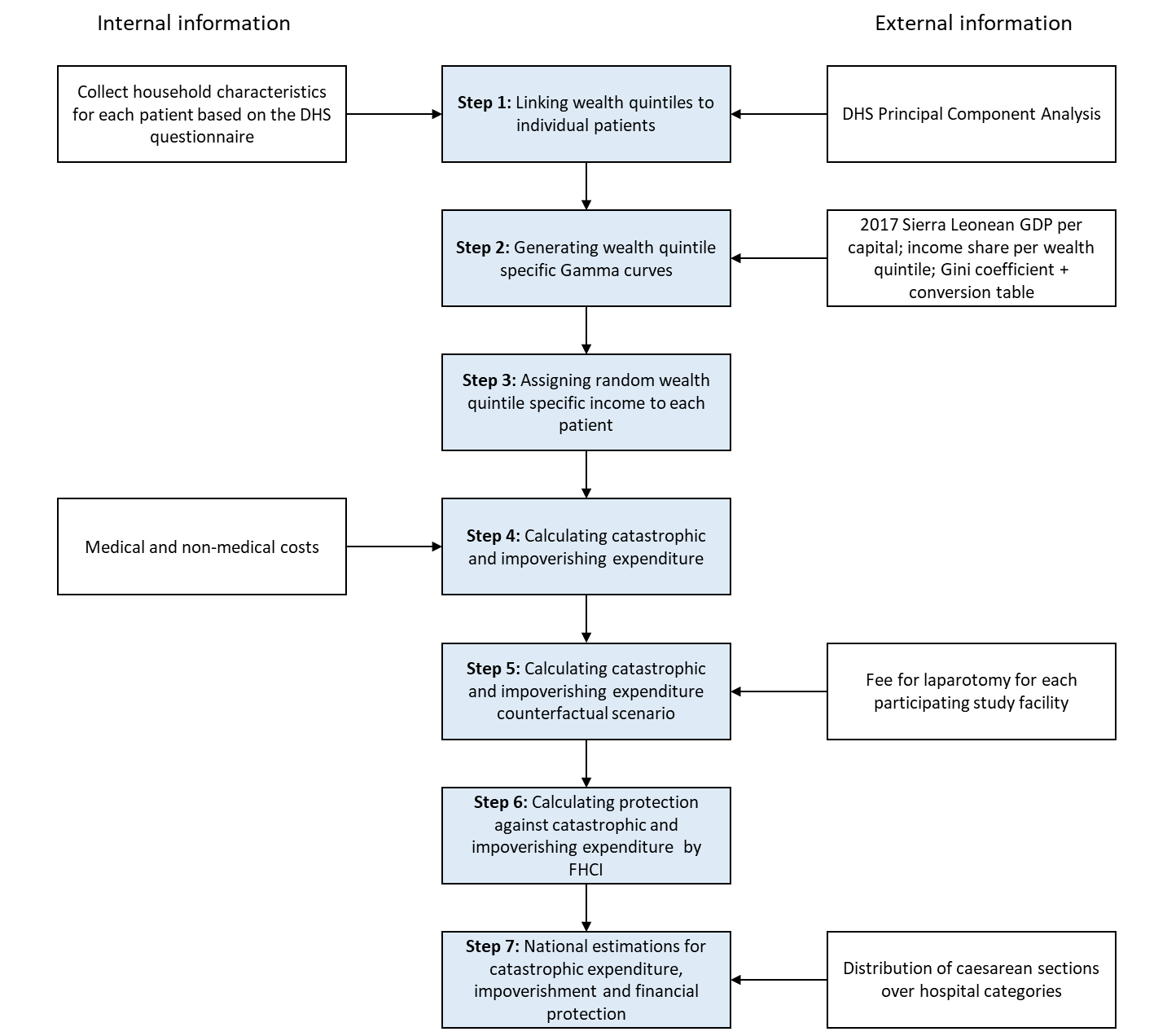


DHS = Demographic and Health Survey; FHCI = Free Health Care Initiative.

**Step1: Linking wealth quintiles to individual patients**

Wealth quintiles are a relative measure of how wealth is distributed in a population and reflects economic status. A predefined population is divided in five equal sized groups based on their assets. Data on such assets are typically obtained within Demographic and Health Surveys (DHS) whereby information is collected from randomized cluster samples during household visits. Visited households are surveyed about assets such as mobile telephone, motorbike, wardrobe, type of floor and roof, etc. These data are subsequently analysed in a Principal Component Analysis (PCA) so that the households in five equal sized groups [1]. The PCA determines how each surveyed parameter contributes to the wealth score and defines the cut-off value for each wealth score for the different quintiles. For each DHS, the PCA is published on the website of the DHS program (https://dhsprogram.com/topics/wealth-index/Wealth-Index-Construction.cfm).

We collected household characteristics data, during the home visits one month after caesarean section, using the 2013 Sierra Leone DHS (Appendix I). Wealth scores were determined by applying the 2013 Sierra Leone DHS PCA, as published by the DHS program. Wealth scores were translated into wealth quintiles by applying cut-off from the PCA.

**Step2: Generating wealth quintile specific gamma curves**

Income distribution can be mathematically described as a gamma (*γ*) distribution [2–4]. The formula of the gamma distribution is as follows, where *α* and *β* are positive parameters that determine the shape of the curve and *Γ* represents the gamma function.

$$f\left( x,\alpha,\beta\right)=\frac{1}{\text{Γ}\left( \alpha\right)\beta^{\alpha}}x^{\alpha-1}e^{-\frac{x}{\beta}}$$

The shape parameter *α* is set with reference to the countries Gini coefficient applying the conversion table described by Shrime et al [3]. For this study we used the 2011 Sierra Leone Gini coefficient of 34.0 (data.worldbank.org) transferred in to the gamma-shape parameter 2.506.

The parameter *β* is the mean income divided by *α*. For wealth quintile specific income distributions, the mean wealth quintile specific mean income is taken as parameter *β*. In this study we have taken the wealth quintile specific GDP per capita [5]. Wealth quintile specific GDP per capita was calculated by taking the 2017 GDP per capita, PPP (constant 2011 international $) times the wealth quintile specific income share. Table 1. Present the quintile specific income. Figure 2 show the wealth quintile specific income distribution curves.

*Table 1. Quintile specific income*

| Wealth quintiles | Income share* | Ratio compared to mean | Mean Income (I$) |
| --- | --- | --- | --- |
| I (poorest) | 7.9% | 0,40 | 554 |
| II | 11.9% | 0,60 | 835 |
| III | 15.8% | 0,79 | 1109 |
| IV | 21.9% | 1,10 | 1537 |
| V (richest) | 42.4% | 2,12 | 2976 |
| Total | 100% | 1.00 | 1404 |

*Figure 2.* *Wealth quintile specific gamma curves*


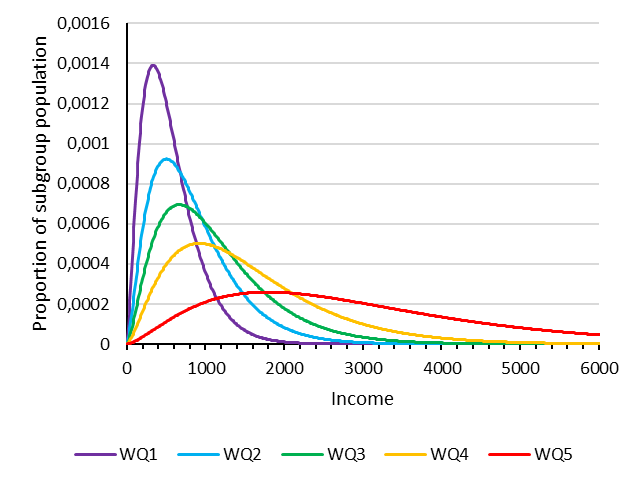


**Step 3: Assigning random wealth quintile specific income to each patient**

Wealth quintile specific gamma curves were changed into cumulative curves (Figure 3). A random selection step was utilised to assign individual annual household incomes to each patient [6]. This was done by changing the wealth quintiles into frequency curves. Microsoft Excel was used to draw a random number between 0 and 1 for each individual patient. This number was consecutive translated into a quintile specific income, based on the cumulative wealth quintile specific curves.

*Figure 3. Cumulative proportion and Income*

**
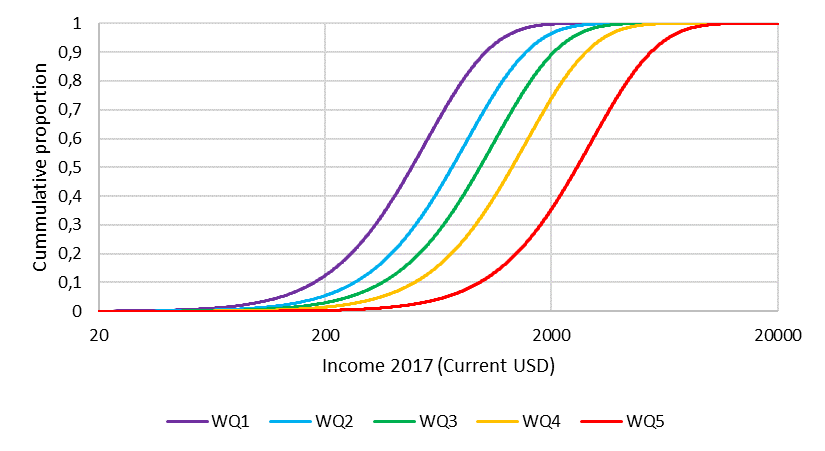
**

**Step 4: Calculating catastrophic and impoverishing expenditure**

All medical and non-medical expenses were recorded in the local currency, Sierra Leone Leones (SLL). Medical costs included: admission fee, consultation fee, medication, other fees. Non-medical costs included: travel, food and lodging (Appendix II).

All expenses were adjusted for time by dividing by the 2011–2017 SLL deflation correction of 1.69 [5,7].

All expenses of tradeable items (medication and food) were converted to Int$ by applying the 2011 market exchange rate of 4349. All non-tradable expenses (admission, consultation, travel, and lodging) were converted to Int$ using the 2011 purchasing power parity (PPP) conversion rate of 1553 [22,30].

Catastrophic expenditures and impoverishment are two measures to quantify financial hardship. Catastrophic expenditure was defined as total out-of-pocket expenses exceeding a set proportion of annual household income using the internationally established thresholds of 10% and 25%.[8] See the catastrophic expenditure formula below where *c* is the out-of-pocket costs, *t* is the threshold 10% and 25% and *y* is the annual income [3].

$$c\geq t\cdot y$$

Impoverishing expenditure was defined as expenditure that pushed individuals under the poverty line (Int$ 1.90 per day or Int$ 694 per year). See the impoverishing expenditure formula below where *T* is the impoverishment threshold (poverty line), *c* is the out-of-pocket costs, and *y* is the annual income.[3]

$$y-c<T$$

To calculate both the catastrophic expenditure and impoverishment indicators, the annual income and the health-related expenses are required.

**Step 5: Calculating catastrophic and impoverishing expenditure counterfactual scenario**

To study the impact of the Free Health Care Initiative (FHCI), a counterfactual scenario was generated, in which patients would have to pay for their caesarean section. As patient fees for caesarean sections have been abolished since 2010, the patient fee for a laparotomy (ranging from Int$ 210 and Int$ 631, dependent on the hospital) was used as a proxy for the cost of a caesarean section and added to total expenses to simulate a situation without the FHCI. The price of a laparotomy was selected as a proxy for the price of a caesarean section as the fees for these two procedures were the same (Le 200,000) before the implementation of the FHCI and are still comparable in the private for-profit health sector in Sierra Leone, which does not participate in the FHCI [9].

**Step 6: Calculating protection against catastrophic and impoverishing expenditure by FHCI**

The impact of the FHCI in terms of protection against catastrophic expenditure is defined as the number of patients who would have faced catastrophic expenditure without the FHCI minus the number who actually faced catastrophic expenditure divided by the number who would have faced catastrophic expenditure without the FHCI.

$$\frac{n_{CE}-n_{CEF}}{n_{CE}}$$

Whereby *n_CE_* is the number patients who would have faced catastrophic expenditure without the existence of the Free Health Care Initiative and *n_CEF_* is the number who actually faced catastrophic expenditure.

The same calculations were performed for impoverishment.

$$\frac{n_{IE}-n_{IEF}}{n_{IE}}$$

Whereby *n_IE_* is the number patients who would have faced impoverishing expenditure without the existence of the Free Health Care Initiative and *n_CEF_* is the number who actually faced impoverishment.

**Step 7: National estimates for catastrophic expenditure, impoverishment and financial protection**

In Sierra Leone, a nation-wide surgical volume mapping was performed by Lindheim *et al*. [9]. From this 3-month sample we extracted number of caesarean sections for each hospital category:

| Category | Facilities; n | Caesarean sections; n (%) |
| --- | --- | --- |
| District hospitals | 12 | 651 (33.8%) |
| Regional hospitals | 3 | 404 (21.0%) |
| Tertiary hospital | 1 | 460 (23.9%) |
| Private non-profit hospitals | 12 | 342 (17.8%) |
| Private for-profit hospitals | 5 | 67 (3.5%) |
| Total | 33 | 1924 (100.0%) |

As the private for-profit hospitals contributed for less than 4% of the total volume of caesarean sections and none of these hospitals were included in the study, this category was not included in the weighting. The following weighting factors were applied:

| Category | Weighting factor |
| --- | --- |
| District hospitals | 0.351 |
| Regional hospitals | 0.218 |
| Tertiary hospital | 0.248 |
| Private non-profit hospitals | 0.184 |

$${CE}_{nat}={CE}_{DH}\times W_{DH}+{CE}_{RH}\times W_{RH}+{CE}_{TH}\times W_{TH}+{CE}_{PNP}\times W_{PNP}$$

Whereby *CE_nat_* is the the national estimate for catastrophic expenditure. *CE_DH_*, *CE_RH_*, *CE_TH_*, and *CE_PNP_* are the wealth quintile specific catastrophic expenditures for district, regional, tertiary and private non-profit hospitals. *W_DH_*, *W_RH_*, *W_TH_* and *W_PNP_* are the weighing factors for district, regional, tertiary and private non-profit hospitals.

The same method was used for national estimates for impoverishing expenditure and for the counter factual scenario. National estimates for financial protection were made based on the formula in step 6.

**References**

1. Rutstein SO. Steps to constructing the new DHS Wealth Index. 2014. Available: https://dhsprogram.com/programming/wealth index/Steps_to_constructing_the_new_DHS_Wealth_Index.pdf

2. Salem ABZ, Mount TD. A Convenient Descriptive Model of Income Distribution: The Gamma Density. Econometrica. 1974;42: 1115–1127.

3. Shrime MG, Dare A, Alkire BC, Meara JG. A global country-level comparison of the financial burden of surgery. Br J Surg. 2016;103: 1453–1461. doi:10.1002/bjs.10249

4. Shrime MG, Verguet S, Johansson KA, Desalegn D, Jamison DT, Kruk ME. Task-Sharing or Public Finance for Expanding Surgical Access in Rural Ethiopia: An Extended Cost-Effectiveness Analysis. Essential Surgery: Disease Control Priorities, Third Edition (Volume 1). 2015. Available: http://www.ncbi.nlm.nih.gov/pubmed/26740996

5. The World Bank Group. World Bank Open Data. [cited 9 May 2018]. Available: https://data.worldbank.org

6. Scott JW, Raykar NP, Rose JA, Tsai TC, Zogg CK, Haider AH, et al. Cured into Destitution. Ann Surg. 2018;267: 1093–1099. doi:10.1097/SLA.0000000000002254

7. Shrime MG, Alkire BC, Grimes C, Chao TE, Poenaru D, Verguet S. Cost-Effectiveness in Global Surgery: Pearls, Pitfalls, and a Checklist. World J Surg. 2017;41: 1401–1413. doi:10.1007/s00268-017-3875-0

8. Margolis D, Rosas N, Abubakarr T, Turay S. Findings from the 2014 Labor Force Survey in Sierra Leone. Washington DC, USA; 2016.

9. Lindheim-Minde BB, Gjøra A, Bakker JM, van Duinen AJ, van Leerdam D, Smalle IO, et al. Changes in surgical volume, workforce and productivity in Sierra Leone between 2012 and 2017. Surgery. 2021. doi:10.1016/j.surg.2021.02.043

**Appendix I. Sierra Leone 2013 DHS Questionnaire – Household characteristics section**

**
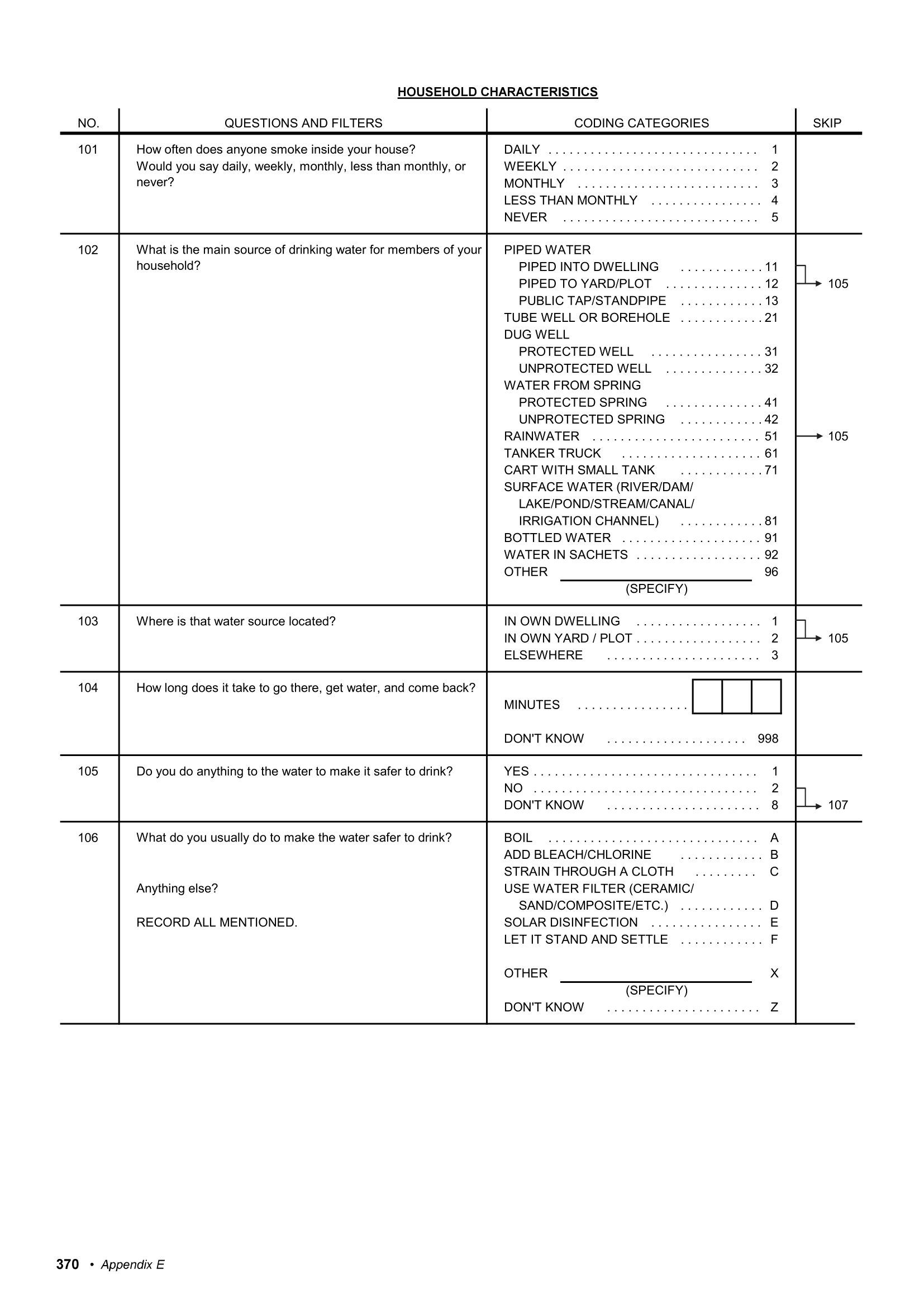
**

**
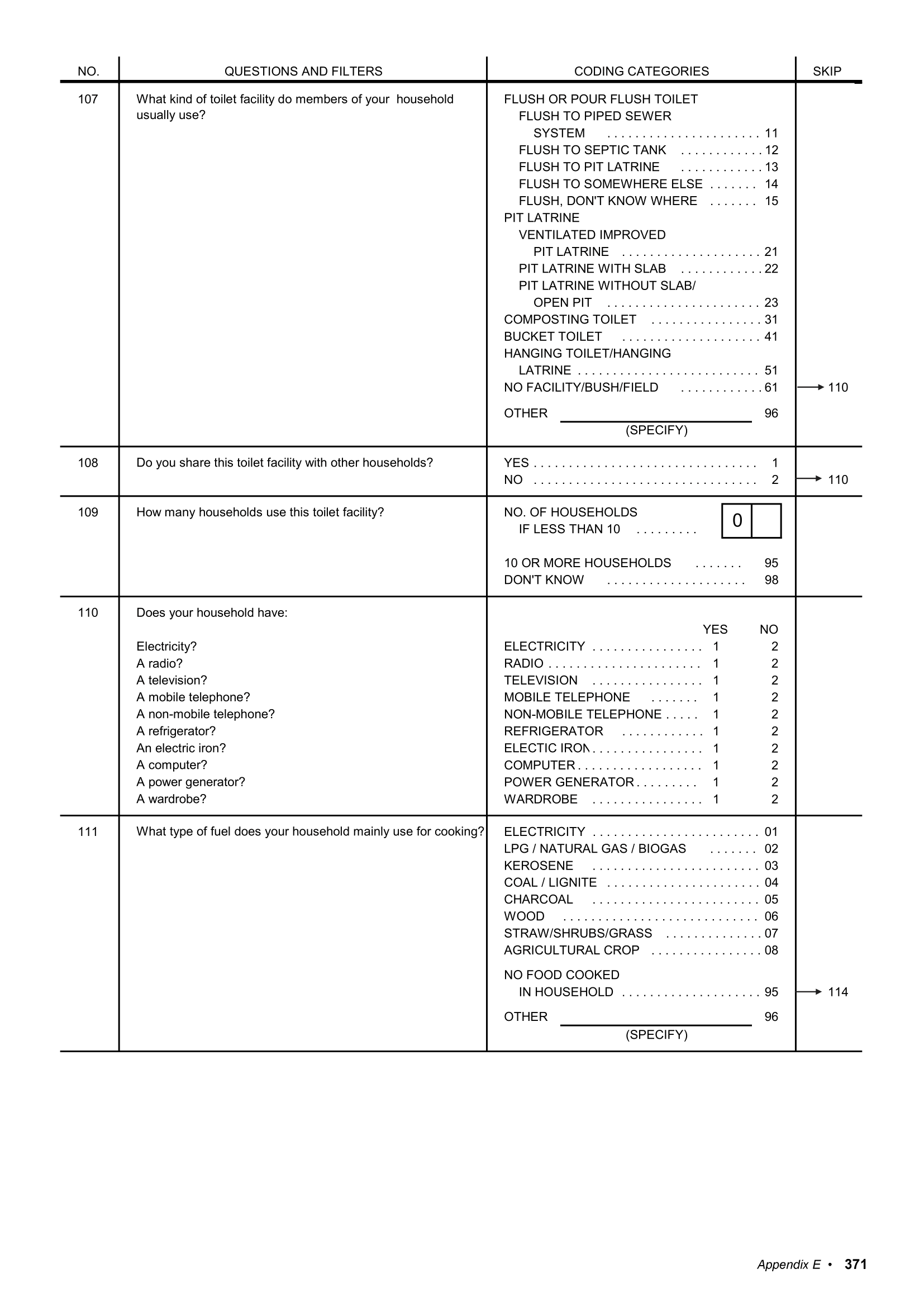

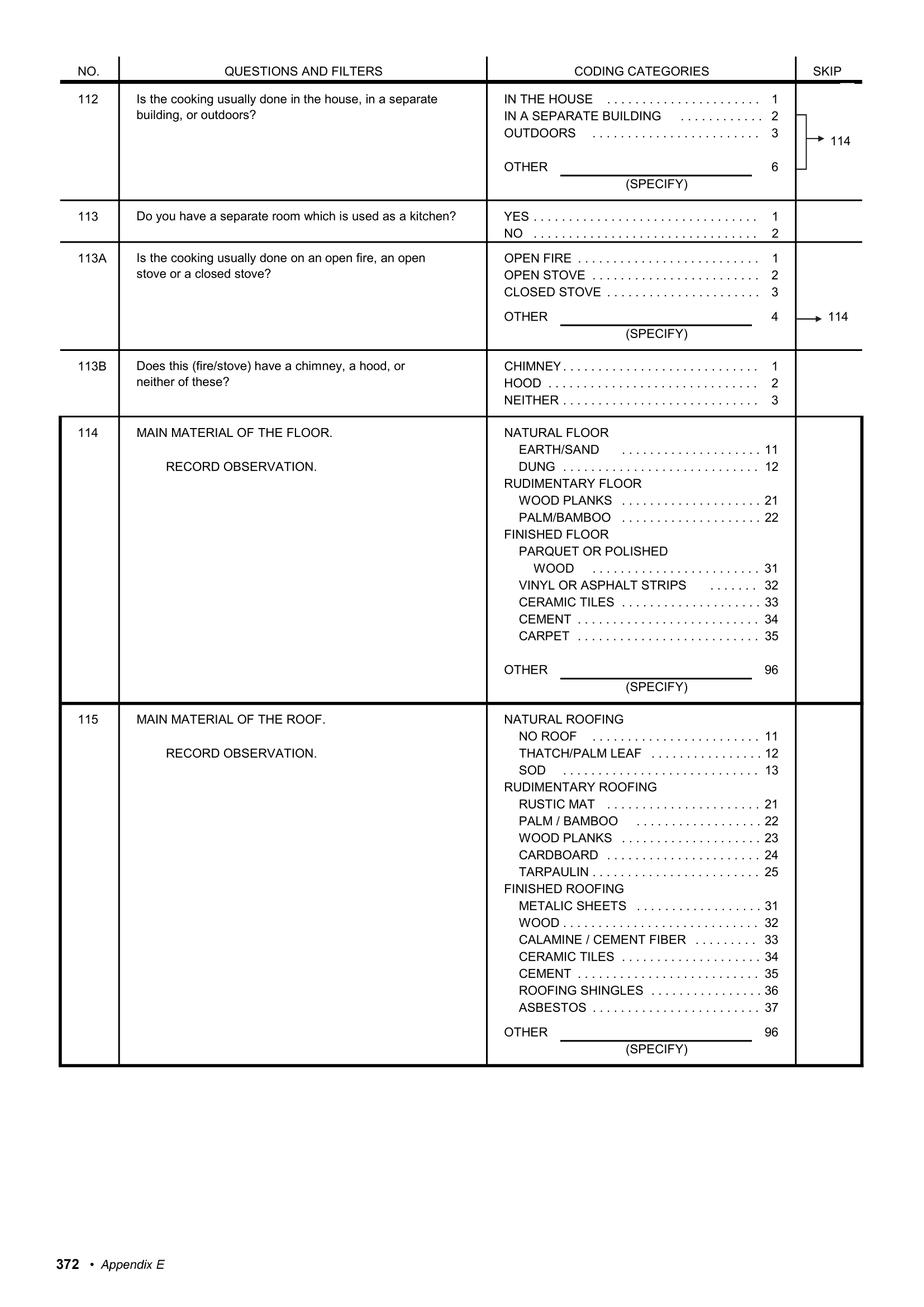

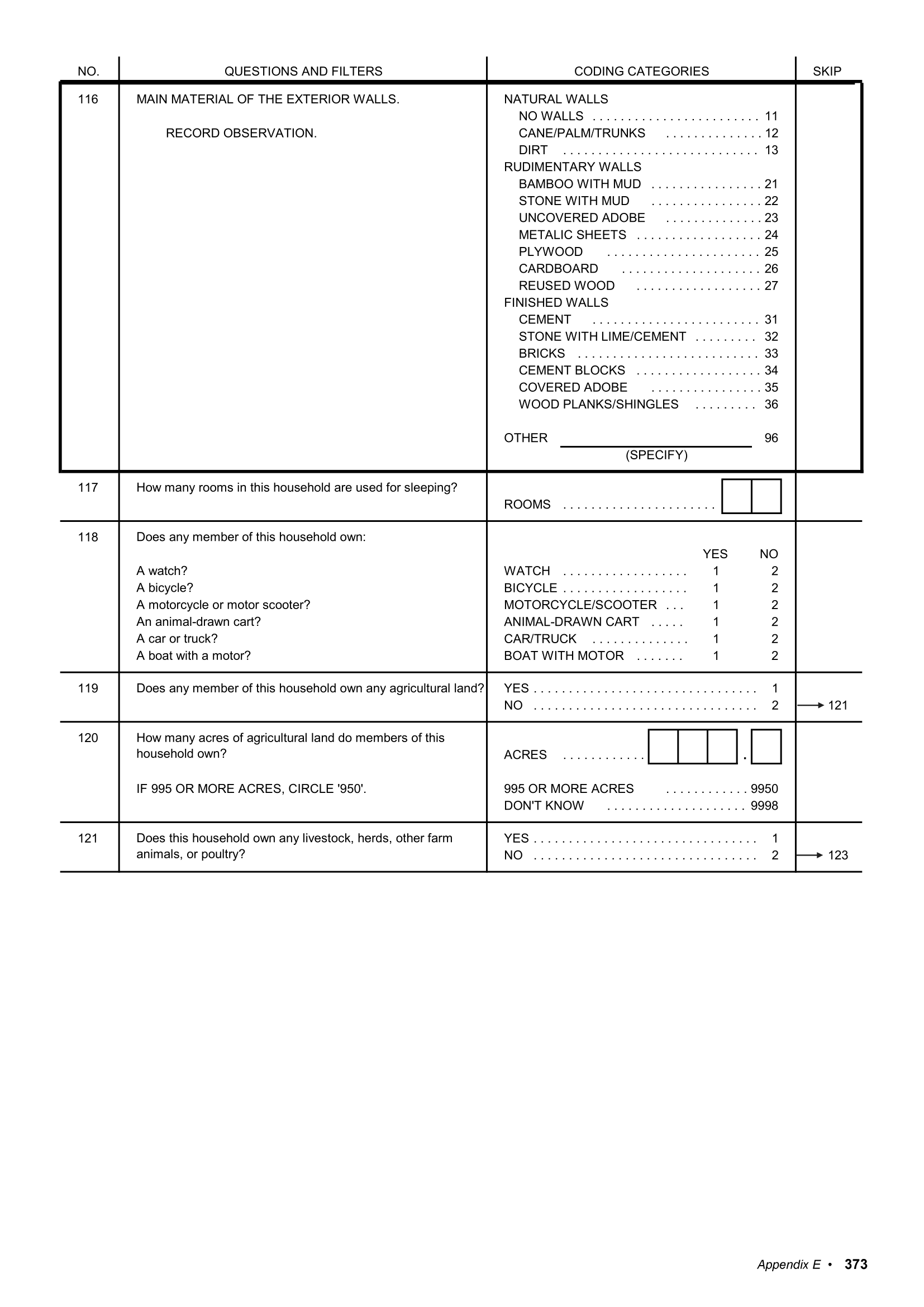

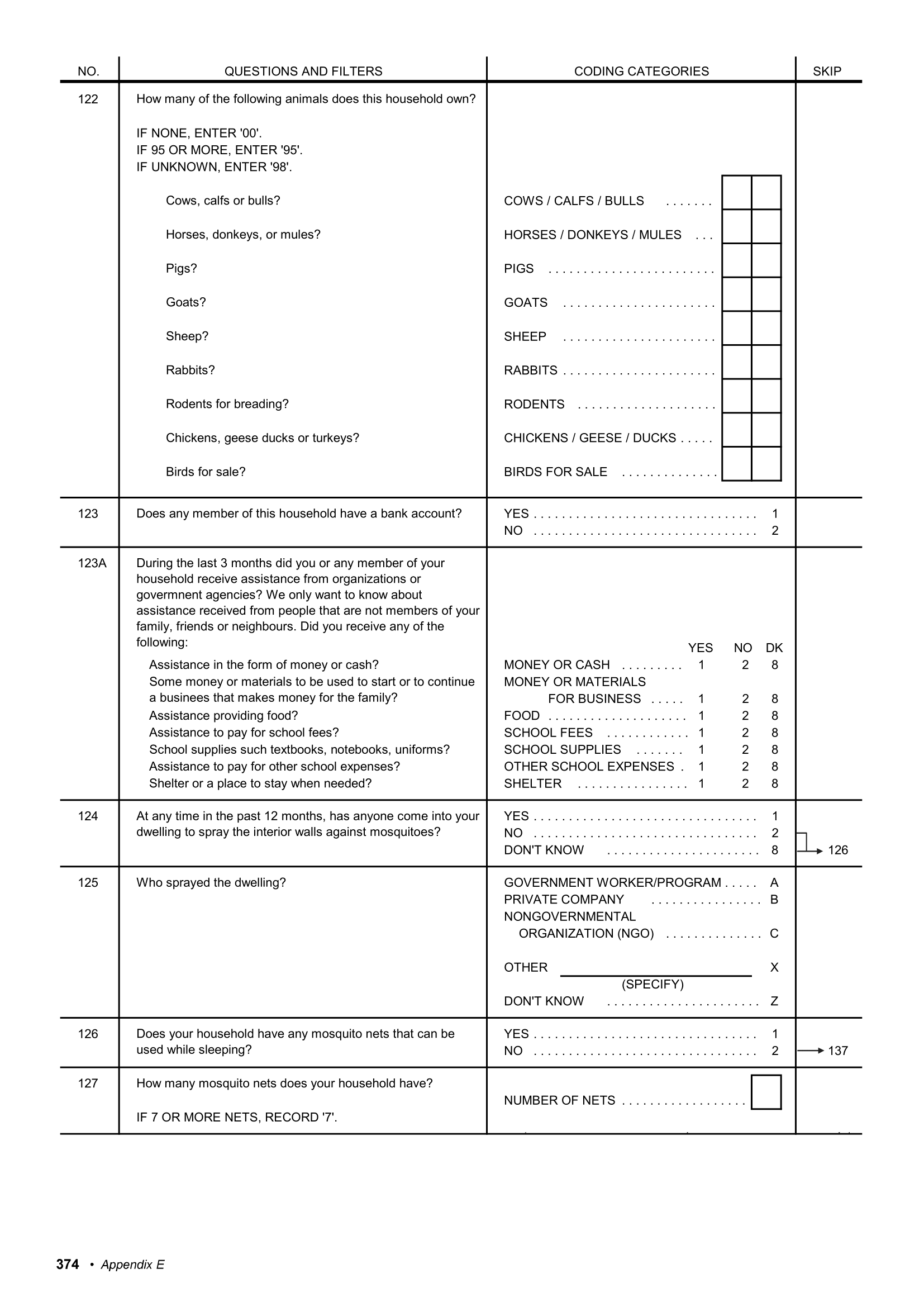

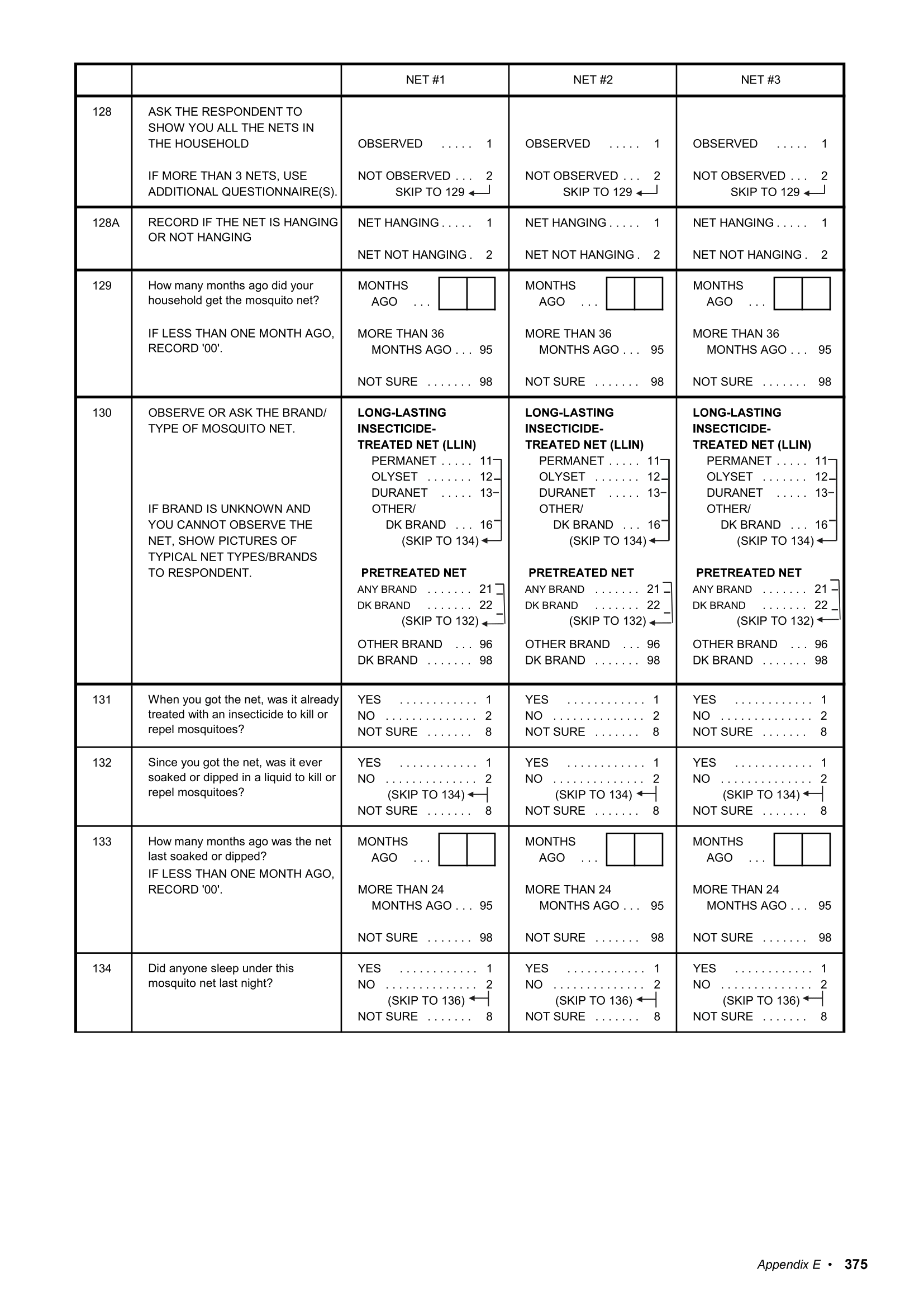

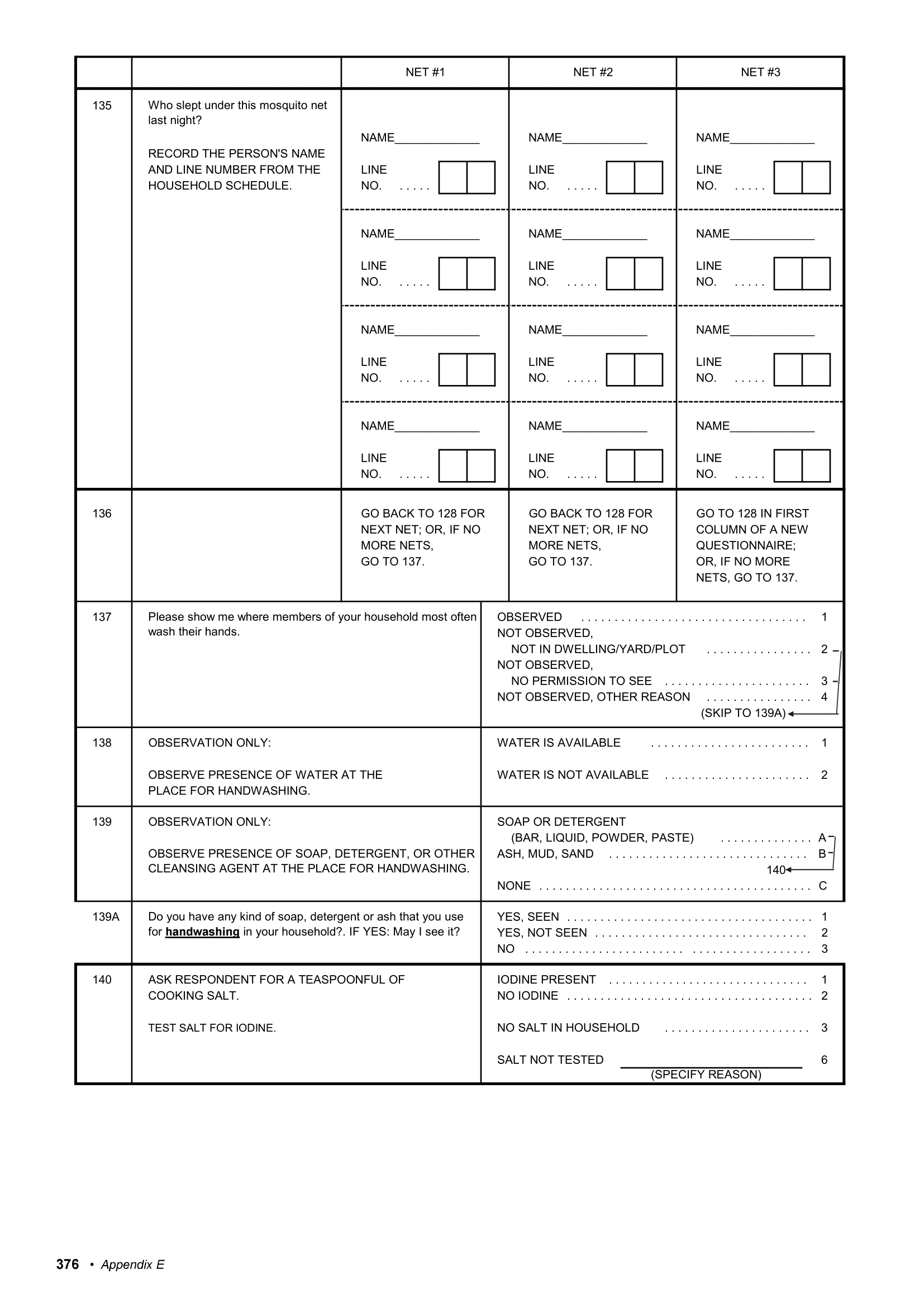
**

**Appendix II. Survey questions regarding expenses**
